# Fourth Disease

**Published:** 1901-12-28

**Authors:** 


### "FOURTH DISEASE."

As is well known, German measles may occur in two types—the morbilliform and the scarletiniform—and that the morbilliform cases are often classed as ordinary measles, while cases of the scarletiniform type are commonly enough regarded as scarlet fever. Dr. Clement Dukes, however, would restrict the term rubella to cases of the morbilliform type, holding that those of scarletiniform type constitute a separate and independent malady which he describes as "fourth disease." This "fourth disease" much resembles scarlet fever in many points, but it differs from it in that it is not dangerous to life and that its period of incubation is much longer than is the case with scarlet fever. A considerable amount of correspondence has taken place lately as to whether this disease really exists as an independent malady, and although Dr. Clement Dukes still holds firmly to his opinion, strong arguments have been advanced against his view. It would seem, indeed that the question must in the end hinge, on the one hand, upon whether or no these scarletiniform cases of rubella which constitute "fourth disease" protect from future attacks of scarlet fever or of German measles, and, on the other, upon whether attacks of these diseases protect from "fourth disease," and of this the evidence from the great fever hospitals gives neither proof nor corroboration. Dr. Washbourn, knowing well how often people are sent into hospitals as the result of mistaken diagnosis, says in a letter to a contemporary that if a disease so closely resembling scarlet fever really existed cases must be admitted into the scarlet fever wards of hospitals where they would from time to time give rise to limited epidemics of "fourth disease." But Dr. Foord Caiger has stated that in his experience at the South Western Hospital he has never met with such an occurrence, and Dr. Washbourn's own experience at the London Fever Hospital tells the same tale; nor has he ever heard of any such epidemic arising in any other hospital. And there the matter stands at present.

